# A Rare Combination: Congenital Adrenal Hyperplasia Due To 21 Hydroxylase Deficiency and Turner Syndrome

**DOI:** 10.4274/Jcrpe.767

**Published:** 2012-12-19

**Authors:** Havva Nur Peltek Kendirci, Zehra Aycan, Semra Çetinkaya, Veysel Nijat Baş, Sebahat Yılmaz Ağladıoğlu, Aşan Önder

**Affiliations:** 1 Dr. Sami Ulus Maternity Children s Health and Disease Training and Research Hospital , Pediatric Endocrinology Clinic, Ankara, Turkey

**Keywords:** Turner syndrome, congenital adrenal hyperplasia, sex differentiation disorder, karyotyping

## Abstract

A combination of Turner syndrome (TS) and classical congenital adrenal hyperplasia (CAH) is rare. A one-day-old newborn was referred to our hospital with ambiguous genitalia. The parents were third-degree relatives. The infant’s weight was 3350g (50-75p), and the head circumference was 34.5cm (50p). The gonads were nonpalpable. Presence of a 3 cm phallus, one urogenital opening into the perineum, and incomplete labial fusion were identified. Laboratory tests revealed a classical type of CAH due to 21-hydroxylase deficiency. Karyotyping revealed a 45X0(35)/46XX(22) pattern with negative sex-determining region Y (SRY) on gene analysis. At the most recent follow-up visit, the patient appeared to be in good health - her height was 70.4 cm [-1.5 standard deviation (SD)] and her weight was 9.8 kg (0.3 SD). She was receiving hydrocortisone in a dose of 10 mg/m^2^/day, fludrocortisone acetate in a dose of 0.075 mg/day, and oral salt of 1 g/day. System examinations were normal. The patient’s electrolyte levels were found to be normal and she was in good metabolic control. The findings of this patient demonstrate that routine karyotyping during investigation of patients with sexual differentiation disorders can reveal TS. Additionally, signs of virilism should always be investigated at diagnosis or during physical examinations for follow-up of TS cases. [i][/i]SRY analysis should be performed primarily when signs of virilism are observed. CAH should also be considered in patients with negative [i]SRY[/i].

**Conflict of interest:**None declared.

## INTRODUCTION

Congenital adrenal hyperplasia (CAH) describes a group of autosomal recessive disorders characterized by enzyme defects in the steroidogenic pathways. 21-hydroxylase (21-OH) is the most common of these enzyme deficiencies and constitutes up to 95% of cases. The classical forms of 21-OH deficiency occur in about 1 in 14 000 persons (1).

Turner syndrome (TS) is accompanied by problems such as short stature, amenorrhea, skeletal and lymphatic abnormalities, hearing loss, aortic coarctation or stenosis, renal abnormalities, thyroiditis, metabolic abnormalities such as carbohydrate intolerance, ovarian insufficiency, and infertility (2). The incidence of abnormalities in the sex chromosomal karyotype resulting in the loss of all or part of an X chromosome has been variously reported as 1:2000 to 1:5000 in live-born phenotypic females (2,3). Diagnosis of CAH is difficult in females with TS, because it shares common clinical features like short stature, amenorrhea, and infertility. Moreover, the combination of TS and classical CAH is rarely reported in literature (4). In this article, we present a newborn patient who was diagnosed to have CAH and TS concomitantly. 

## CASE REPORTS

**Figure 1:**
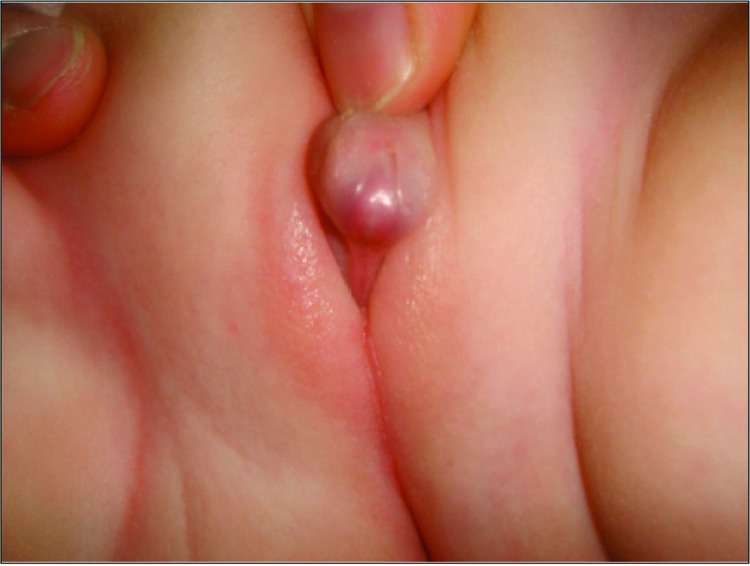
Appearance of the external genitalia in the patient

A one-day-old newborn was referred to our hospital with ambiguous genitalia. According to the medical history, she was born at term to a 17-year old healthy mother from her first gestation and who was delivered by cesarean section due to cephalopelvic disproportion. Birth weight was 3500g. The parents were third-degree relatives. On physical examination, the general status was good, vital signs were stable, weight was 3350g (50-75p), and head circumference was 34.5cm (50p). Genital examination revealed nonpalpable gonads, a 3cm phallus, cordae, one urogenital opening into the perineum, and an incomplete labial fusion (Figure 1). In the laboratory evaluation, blood count, urinalysis, renal and liver function tests were normal. Biochemistry revealed a glucose level of 52 mg/dL, sodium (Na): 141.2 mEq/L, potassium (K): 4.6 mEq/L, adrenocorticotropic hormone (ACTH): 186 pg/mL, cortisol: 4.2 μg/dL, 17-hydroxyprogesterone (17-OHP): 30.4 ng/mL, dehydroepiandrosterone sulfate (DHEA-S): 129 μg/dL, total testosterone: 776 ng/dL, progesterone: 4.05 ng/mL, aldosterone: 1745.9 pg/mL, and renin activity: 23.6 ng/mL/hour. Pelvic ultrasonography demonstrated a uterus with diameters of 2.3 x 0.9 x 1.2 cm. The right ovary was 0.8x0.7 cm and the left ovary was 0.9 x 0.5 cm. In the gene analysis of CYP21, a homozygote IVS2-13A/C>G mutation was detected. At a standard-dose ACTH test, peak cortisol level was 16.6 μg/dL (<20), while the peak 17-OHP level was 35.1 ng/mL. These results suggested a classical type of CAH due to 21-OH deficiency. Hydrocortisone treatment at the dose of 30 mg/m2/day was hence initiated. On the 10^th^ day of hospitalization, the baby became feverish and the general status deteriorated. White blood cell count was 18 700x10^3^/μL, CRP was 33.9 mg/dL, Na 129 mEq/L, and K 6.8 mEq/L. A diagnosis of nosocomial septicemia and related adrenal crisis was considered. The hydrocortisone dose was doubled; fludrocortisone acetate, 1.5 g/day orally and an intravenous antibiotic were also added to the treatment. After recovery from adrenal crisis and septicemia, the patient was discharged on the 27^th^ postnatal day. TS was diagnosed with karyotype analysis which showed a pattern of 45X0(35)/46XX(22), while the sex-determining region Y (SRY) gene analysis was found to be negative. Renal ultrasonography and echocardiography, performed as part of routine investigations in TS patients, revealed no pathology. Cliteroplasty was performed when the patient attained 6 months of age. During the last follow-up visit, when she was one-year-old, her general status was reported to be good. Her height was 70.4cm [-1.5 standard deviation (SD)], weight was 9.8kg (0.3 SD), and system examinations were normal. The patient’s electrolyte levels were found to be normal and she was in good metabolic control while taking hydrocortisone in a dose of 10 mg/m^2^/day, fludrocortisone acetate 0.075 mg/day, and oral salt of 1 g/day.

## DISCUSSION

Six cases with concomitant TS and CAH have been reported in the literature. In 1983, del Arbol et al (5) first reported an 8-year-old girl, with 45,X/46,X,Xq karyotype, a 2.5 cm phallus, and a hormonal profile which was consistent with non-classical CAH. In 1985, Montemayor-Jauregui et al (6) reported a 23-year-old female patient with primary amenorrhea, 3 cm phallus, hirsutism, and 45,X/46XX chromosome pattern. This case was diagnosed as CAH following the detection of high levels of 17-ketosteroids in the 24-hour urine sample. The hormonal profile later returned to normal and menarche commenced following initiation of prednisolone treatment. A 16.9-year-old female patient with 45,X0 karyotype and cliteromegaly is the third case of this unusual combination, which was reported in 1992 by Larizza et al (8). In 1997, Maciel-Guerra et al (3) published a one-year-old patient with a 3cm phallus, penoscrotal hypospadias, and 45X0/46XX karyotype, who was diagnosed as CAH, and who, during the newborn period, was evaluated as male, had nonpalpable gonads with penoscrotal hypospadias, and experienced three adrenal crises. Cohen et al (7) also reported the case of a 28-year-old female patient known to have TS and who was diagnosed as CAH during the oocyte donation process. Lastly, in 2005, Atabek et al (4) reported a patient from Turkey, who had ambiguous genitalia at birth, and who was diagnosed as a case of TS at the age of one year, by detection of the 45,X/46XX karyotype. Presenting for evaluation of signs of pubarche, genital examination of this patient at age 4 years revealed Prader stage 3, a bone age of 8 years, and basal and stimulated 17-OHP levels consistent with a diagnosis of the simple virilising form of CAH (4). As in our case, the majority of previously reported cases were diagnosed as TS while being investigated for ambiguous genitalia. Two cases known to have TS were later diagnosed as CAH.

In a study conducted on 52 Italian TS cases and their relatives, basal and stimulated serum 17-OHP levels were found to be much higher than those in normal controls, and similar hormonal data were encountered also in the relatives of these patients (8). These data were considered to be related to increased adrenal sensitivity to ACTH or to changes in metabolic clearance rate of 17-OHP, as observed in obese patients. However, HLA antigen and haplotype frequencies in the patients and their relatives had a similar distribution in Italian families with 21-OH deficiency. According to the results of this study, the frequency of 21-OH deficiency carriers in TS patients was remarkably higher (21.6%) than that of the general Italian population (8).

Mantovani et al (9) also reported that the frequencies of both abnormal 17-OHP response to ACTH stimulation test and CYP21 gene mutation carriers were prominently higher in patients with TS than in healthy controls. They speculated that while more than 90% of conceptions with 45X0 karyotype normally resulted in spontaneous abortion, certain endocrine signals originating from embryos with decreased 21-OH activities may lead to relaxation of maternal screening, and so provide survival advantage for heterozygote patients with 21-OH deficiencies (9).

Concomitant existence of TS and CAH may be associated with some problems. Firstly, diagnosis becomes difficult because of the presence of some common features like short stature, infertility, and amenorrhea (4). Our case presented with ambiguous genitalia during the newborn period, and during investigation, she was diagnosed as CAH, but later as TS following routine karyotype analysis. Considering the fact that the mean age of TS diagnosis is 10-11 years, diagnosis of patients with common clinical features may be delayed until this age, particularly when signs of virilism are not as prominent as in our case. Moreover, in addition to the classical 45X0 chromosomal structure observed in TS, the karyotype can show a wide spectrum, and the X and Y chromosomes may be accompanied by mosaic monosomy. Virilism can also be observed in TS patients with a Y chromosome. These patients do not only present with ambiguous genitalia but also carry the risk for development of a malignant gonadal tumor (3). Our patient had the mosaic form of TS and presented with features of virilism. However, she had no Y chromosome. Her basal and ACTH-stimulated concentrations of 17-OHP clearly established a diagnosis of the classical form of 21-OH deficiency. Additionally, the CYP21 gene analysis of our patient revealed a homozygous IVS2-13A/C>G mutation.

Another problem of the TS and CAH combination is short stature. Inadequacy of hormone replacement therapy or overtreatment of CAH causes final short stature. However, in TS, there is a tendency for progressive deviation from normal height percentile due to retarded bone age together with decrease in the growth rate. The final heights of patients with concomitant TS and CAH have a tendency to deteriorate due to presence of both diseases (3). While it is possible to obtain good results in CAH patients treated with careful clinical and laboratory follow-up, in TS, growth hormone (GH) treatment initiated at supraphysiological doses and at an early age can also lead to acceptable increases in height, despite the absence of GH deficiency (4). Optimal treatment of TS with GH is suggested to be initiated at an early age (before age 4 years), and high doses are recommended particularly during the first year of treatment (10). The advantage of our case was the diagnosis of TS at a very early age. Moreover, in addition to treatment with appropriate doses of glucocorticoids and mineralocorticoids, GH treatment was initiated when a slowing in growth was later observed.

In conclusion, the findings in this patient show that routine karyotyping during investigations of patients presenting with ambiguous genitalia or with a diagnosis of CAH may reveal the concomitant presence of TS.

Additionally, signs of virilism should always be investigated at diagnosis or during the follow-up of patients with TS. In the presence of signs of virilism, SRY analysis should be performed primarily, and CAH evaluation should be considered in patients with negative SRY.
